# Rapid direct identification of positive paediatric blood cultures by MALDI-TOF MS technology and its clinical impact in the paediatric hospital setting

**DOI:** 10.1186/s13104-019-4861-4

**Published:** 2020-01-06

**Authors:** Waniganeththi Arachchige Manori Piyumal Samaranayake, Suzanne Dempsey, Annaleise R. Howard-Jones, Alexander Conrad Outhred, Alison Margaret Kesson

**Affiliations:** 1 Department of Infectious Diseases and Microbiology, The Children’s Hospital at Westmead, Sydney, Australia; 2 The Marie Bashir Institute of Infectious Diseases and Biosecurity, The University of Sydney, Sydney, Australia; 3 Discipline of Child and Adolescent Health, The University of Sydney, Sydney, Australia

**Keywords:** Direct identification of blood culture, MALDI-TOF mass spectrometry, Clinical impact, Pediatric, Bloodstream infection

## Abstract

**Objective:**

Rapid diagnostic tools are imperative for timely clinical decision making, particularly in bacteraemic patients. This study evaluated the performance of a fast, inexpensive novel in house method for processing positive blood cultures for immediate identification of microorganisms by matrix-assisted laser desorption ionization-time of flight mass spectrometry (Vitek MS bioMérieux). We prospectively analyzed the clinical impact of such method on the management of pediatric patients.

**Result:**

In total, 360 positive blood cultures were included. Among 318 mono-microbial cultures, in-house method achieved correct identification in 270 (85%) cultures to the species level, whilst 43 (13.5%) gave no identification, and 7 (2.2%) gave discordant identifications. Identification of Gram-negative organisms was accurate to both species and genus level in 99% of isolates, and for Gram positives accuracy was 84% to genus and 81% to species level overall, with accuracy of 100% for *Staphylococcus aureus* and *Enterococcus* to the species level. Assessment of the potential impact of direct identification in sixty sequential cases revealed a clear clinical benefit in 35.5% of cases. Benefits included timely antibiotic rationalization, change of medical intervention, and early confirmation of contamination. This study demonstrates a highly accurate in-house method with considerable potential clinical benefits for paediatric care.

## Introduction

Sepsis is associated with high mortality [1]; hence rapid identification of bloodstream pathogens and timely clinical decision making are paramount. Matrix-assisted laser desorption ionization-time of flight mass spectrometry (MALDI-TOF MS) has revolutionized microbiology laboratories with improved workflow for pathogen identification both from solid media and positive blood culture broths [2–4]. Conventional processing requires overnight incubation of the blood culture broth on solid media prior to MALDI-TOF MS analysis, delaying organism identification by 18–24 h.

Recently, several in-house protocols for direct organism identification from positive blood culture broths have been developed [2, 3]. However, these protocols entail either complicated preprocessing methods or short periods of subculture to increase their accuracy [2–8]. As a result, these methods can be tedious and expensive [3], and remain controversial [2]. Performance rates vary, such that there is no consensus on best-practice [2, 9–11]; commercial kits are neither cost-effective nor superior in performance compared to in-house methods [12]. Additionally, most studies to date have focused on adult populations and on the specific Bruker Daltonics Biotyper system [3].

Direct MALDI-TOF MS identification greatly reduces time to pathogen identification [4, 5]. In a single center open-label controlled clinical trial, Osthoff et al. demonstrated significant reductions in time to pathogen identification and admissions to the intensive care unit, but no difference in mortality, length of stay in a rapid identification group compared to routine methods [5]. Cost-savings for the laboratory from direct MALDI-TOF identification have also been documented [2–4]. On this basis, we developed a simple fast economical in-house method for accurate identification of positive pediatric blood culture broths using the Vitek MS (bioMérieux) system. Subsequently, we prospectively evaluated the clinical outcome of such a method on pediatric inpatient care.

## Main text

### Methods: Study setting

This study was conducted at the bacteriology laboratory at the Children’s Hospital at Westmead, Australia from November 2018 to November 2019. All positive blood culture broths were processed routinely alongside the direct identification protocol for performance comparison.

#### Routine method

Blood was taken with aseptic technique, directly inoculated into aerobic (BacT/Alert FA plus), and/or anaerobic bottles (BacT/Alert FN plus). Bottles were loaded onto the BACT/ALERT^®^ VIRTUO^®^ instrument (bioMérieux) for incubation up to 5 days, or until they signaled as positive. All positive blood cultures were analyzed by Gram stain and subjected to subculture on solid media (blood, MacConkey, chocolate and anaerobic blood agar) then incubated at 35 ± 1 °C in a 5% CO_2_ atmosphere for 18–24 h. Conventional identification methods included biochemical and automated platforms (Vitek II, bio-Mérieux and Vitek MS, bioMérieux), depending on the organism isolated. Discordant identifications were further characterized by 16S rRNA gene sequencing at a reference laboratory when identification was deemed to be clinically relevant.

#### In-house rapid identification protocol

For each positive culture, 1.5 ml of positive blood culture broth was centrifuged at 500 rpm (23*g*) for 2 min to separate erythrocytes from the bacterial cells. 50 µl of Triton (10×) was added to the supernatant followed by gentle mixing and centrifugation for 1 min at 13,000 rpm (15,900*g*). The pellets were re-suspended in 1.5 ml distilled water and centrifuged for 1 min at 13,000 rpm (15,900*g*). 50 µl of formic acid (70%) and 50 µl of acetonitrile were added to the pellet and mixed, then centrifuged for 1 min at 13,000 rpm (15,900*g*). Following this, 1 µl of supernatant was spotted in triplicate onto a target slide and air-dried. Each spot was covered with 1 µl of an alpha-cyano-4-hydroxycinnamic acid matrix for MALDI-TOF MS analysis.

#### MALDI-TOF MS analysis

The target plate was analyzed by the Vitek^®^ MS V3.2 bioMérieux system and matched against a library of data representing 1316 taxa. This analysis returned a result with the best identification match along with a confidence percentage from 0 to 99.9%. A 95 to 99.9% confidence reading on a minimum of one spot was considered high at the species level while 90–94% confidence was considered high at the genus level. If the confidence score was ≥ 50–94%, the result was recorded as ‘genus-level’ if there was a choice of 2–4 organisms all within the same genus. However, if the organisms were distributed between different genera, no valid identification was recorded.

#### Clinical data collection

A prospective clinical impact analysis was conducted over 7 weeks between August and September 2019. For all positive blood cultures identified over this period, a clinical impact assessment was performed against three categories: (1) change in antimicrobials, (2) change in intervention or (3) infection control impacts. Clinical data pertaining to these outcomes, as well as clinical diagnosis and likely significance of a positive culture, were recorded on day one (day of Gram stain result) and day two (day of formal organism identification) after collection of the index culture. Cases where rapid MALDI-TOF gave organism identification were considered as having the potential to change clinical management on day one.

#### Statistical analysis

The identification results of the in-house method were compared with that of the routine identification method. The correct identification rate was recorded as the number of isolates that were correctly identified by routine methods divided by the overall number of isolates in sub-groups. Sensitivity, specificity, positive predictive values and negative predictive values to genus level were calculated to determine diagnostic accuracy using SPSS version 17.

### Results

A total of 360 positive pediatric blood culture broths were collected over the study period. Of these, 308 were from aerobic and 52 from anaerobic bottles. There were 320 mono-microbial infections, and 40 poly-microbial infections. There were two Gram-positive bacteria that were identified only by 16S rRNA gene sequencing and these were excluded from the analysis, Table 1 presents microorganism identification results of the positive mono-microbial blood cultures that were analyzed by the routine method and in-house protocol.

**Table 1:** List of the microorganisms identified by routine method and direct in house method

List of microorganisms identified by routine methods/number of isolates	Number of isolates identified by MALDI-TOF MS at the species level	Number of isolates identified by MALDI-TOF MS at the genus level only	Isolates not identified by MALDI-TOF MS	Isolates with discordant results	Sensitivity (%)	Specificity (%)	PPV (%)	NPV (%)
*Staphylococcus aureus* (36)	36	–	–		100	100	100	100
*Staphylococcus epidermidis* (46)	45	1	–		82.1	97.9	93.3	94.2
*Staphylococcus hominis* (9)	8	–	1					
*Staphylococcus haemolyticus* (2)	2	–	–					
*Staphylococcus capitis* (3)	2	–	1					
*Staphylococcus cohnii* (1)	–	–	1					
*Staphylococcus simulans* (2)	2	–	–					
*Kocuria kristinae* (1)	1	–	–					
*Dolosigranulum pigrum* (1)	–	–	1					
*Rothia mucilaginosa* (2)	2	–	–					
*Micrococcus luteus* (16)	7	–	9	*L. monocytogenes* (2) *L. adecarboxylata* (2) *P. baroniae* (1)				
*Micrococcus terreus* (1)	–	–	1					
Total GPC resembling Staphylococcus (120)	105	1	14		88.3	97.5	95.5	93.2
*Streptococcus agalactiae* (7)	6	–	1					
*Streptococcus pneumoniae* (6)	5	–	1					
*Streptococcus mitis/oralis group* (9)	5	–	4					
*Streptococcus parasanguinis* (2)	2	–	–					
*Streptococcus sanguinis* (1)	1	–	–					
*Streptococcus intermedius* (1)	1	–	–					
Other Viridans group of Streptococci (6)	–	2	4					
*Streptococcus constellatus* (1)	–	–	1					
*Granulicatella adiacens* (1)	1	–	–					
*Abiotrophia defective (2)*	1	–	–	*L. adecarboxylata* (1)				
*Enterococcus faecalis* (23)	23	–	–		100	100	100	100
*Enterococcus faecium* (2)	2	–	–					
Total GPC resembling Streptococcus (61)	47	2	12		80.3	99.6	98	95.5
*Clostridium perfringen* (2)	2	–	–					
*Clostridium septicum* (1)	1	–	–					
*Bacillus cereus* (5)	4	–	1					
*Bacillus megaterium* (1)	1	–	–					
*Bacillus altitudinis/pumilus* (1)	1	–	–					
*Brevibacterium luteolum* (1)	1	–	–					
*Bacillus simplex* (1)	1	–	–					
*Bacillus gibsonii* (1)	1	–	–					
*Dermabacter hominis* (1)	–	–	1					
*Actinomyces viscosus* (1)	–	–	1					
*Curtobacterium flaccumfaciens* (1)	–	–	1					
*Dietzia cinnamea* (1)	–	–	1	*S. cornosus* (1)				
*Corynebacterium aurimucosum* (1)	–	1	–					
*Corynebacterium* spp. (2)	–	–	2					
Total Gram positive rods (20)	12	1	7		65	99.7	92.9	97.7
*Escherichia coli* (29)	29	–	–					
*Klebsiella pneumoniae* (11)	11	–	–					
*Klebsiella oxytoca* (2)	2	–	–					
*Enterobacter cloacae* complex (22)	21	–	1					
*Salmonella enterica* subsp. *enterica* (14)	14	–	–					
*Serratia marcescens* (4)	4	–	–					
*Morganella morganii* (1)	1	–	–					
*Pseudomonas aeruginosa* (9)	9	–	–					
*Acinetobacter radioresistans* (1)	1	–	–					
*Acinetobacter lwoffii* (1)	1	–	–					
*Acinetobacter ursingii* (1)	1	–	–					
*Acinetobacter baumannii* (1)	1	–	–					
*Acinetobacter johnsonii* (1)	1	–	–					
*Stenotrophomonas maltophilia* (7)	7	–	–					
*Neisseria meningitides* (1)	1	–	–					
*Moraxella catarrhalis* (2)	2	–	–					
Total Gram negatives (107)	106	–	1		99	100	100	99.5
*Candida albicans* (1)	–	–	1					
*Candida parapsilosis* (9)	–	–	9					
Total yeast (10)	–	–	10					

*MALDI-TOF* matrix assisted laser desorption ionization-time of flight, *PPV* positive predictive value, *NPV* negative predictive value

#### Performance of in house method

As shown in Table 1, 99% (106/107) of Gram-negative bacteria were identified by the direct method to species level. Gram-positive organisms were accurately identified at a lower rate of 84% (168/201) to the genus level and 81% (164/201) to species level, but this varied greatly by taxa. Identification to the species level was less accurate for coagulase-negative staphylococci (82%) and for Gram-positive rods (65%). Overall, positive predictive values for the direct identification of both Gram-positive and Gram-negative bacteria from mono-microbial blood culture broths to genus level were 96% and 100% respectively. None of the yeast isolates were identified by this method.

Forty blood cultures were confirmed by routine methods to be composed of 2 or 3 bacterial species. Of the 40 poly-microbial blood cultures, direct MALDI-TOF MS correctly reported one of the species present in 23 (58%) of cultures. Identification rates increased to 34 (85%) when the confidence score was lowered to 50–99.9%. The in-house method identified all organisms from 5/40 (13%) poly-microbial blood culture bottles, with triplicate analysis enabling increased accuracy in these mixed organism samples.

#### Clinical outcome

Of the 60 individual patient cases reviewed, 32 were females and 28 were males. The majority (42) were between 1 month to 6 years old. Direct identification yielded a genus or species in 88% (53/60) of cultures. Six specimens did not yield organism identification, and one was a discordant result. Twenty-two specimens represented repeat samples from the same patient with the same organism. Therefore, the clinical impact was determined for the remaining 31 patients’ cases. In eleven cases (35.5%) early MALDI-TOF identification would have had clinical benefit (Table 2). In ten cases (32.3%) a change of antimicrobials would have been facilitated, six cases (19.4%) could have received medical and surgical interventions. No cases had the potential for an infection control intervention.

**Table 2:** Summary of cases for which rapid organism identification would have impacted clinical decision making

Case number	Gram stain day one	Clinical context	Antibiotics on day one	Organisms identified by direct MALDI-ToF on day one	Organisms identified from sub-culture day two	Antibiotic change on day two	Medical/surgical intervention
1	GNR	Pyelonephritis	Vancomycin and Gentamicin	*S. maltophilia*	*S. maltophilia*	Cefotaxime and Cotrimoxazole added	Neprostomy tube removed
2	GNR	Febrile neutropenia due to UTI	Piperacillin/Tazobactam and Gentamicin	*E. coli*	*E. coli*	Cefotaxime added. Gentamicin continued	–
3	GNR	Post-operative liver transplant infection	No antibiotics	*Enterobacter cloacae* complex	*Enterobacter cloacae* complex	Imipenem added	Interventions to sterilise line with antibiotic locks
4	GPR	Myonecrosis	Piperacillin/Tazobactam, Clindamycin and Vancomycin	*C. septicum*	*C. septicum*	High dose benzyl penicillin added clindamycin continued; tazocin and vancomycin ceased	Urgent re-exploration of leg as necrotising fasciitis
5	GPCSTR	Intra muscular abscess	Penicillin and Flucloxacillin	*S.parasanguinis*	*S.parasanguinis*	Penicillin continued Flucloxacillin stopped	MRI to rule out septic arthritis
6	GPCSTR	Meningitis	Cefotaxine, Gentamicin and Ampicillin	*S. agalactiae*	*S. agalactiae*	Benzylpenicillin added Cefotaxime, Gentamicin, Ampicillin ceased	–
7	GPCSTA	Congenital heart disease with fever	Vancomycin and Gentamicin	*S. epidermidis*	*S. epidermidis*	Vancomycin continued Gentamicin ceased	–
8	GPCSTA	Febrile neutropenia	Piperacillin/Tazobactam and Vancomycin	*S. epidermidis*	*S. epidermidis*	Vancomycin continued Piperacillin/Tazobactam ceased	CVL removed
9	GPCSTA	Primary immune deficiency with fever	Piperacillin/Tazobactam and Vancomycin	*S. epidermidis*	*S. epidermidis*	Piperacillin/Tazobactam and Vancomycin continued	CVL removed
10	GPCSTA	Acute bronchiolitis	Vancomycin	*S. epidermidis*	*S. epidermidis*	Vancomycin ceased as probable contamination	–
11	GPCSTR	Cerebral palsy	Vancomycin	*S. mitis*	*S. mitis*	Vancomycin ceased as probable contamination	–

*MALDI-TOF* matrix assisted laser desorption ionization-time of flight, *GNR* Gram negative rods, *GPR* Gram positive rods, *GPCSTR* Gram positive Staphylococci, *GPCSTA* Gram positive Streptococci, *CVL* central venous line, *UTI* urinary tract infection

#### Clinical case

**Fig. 1:**
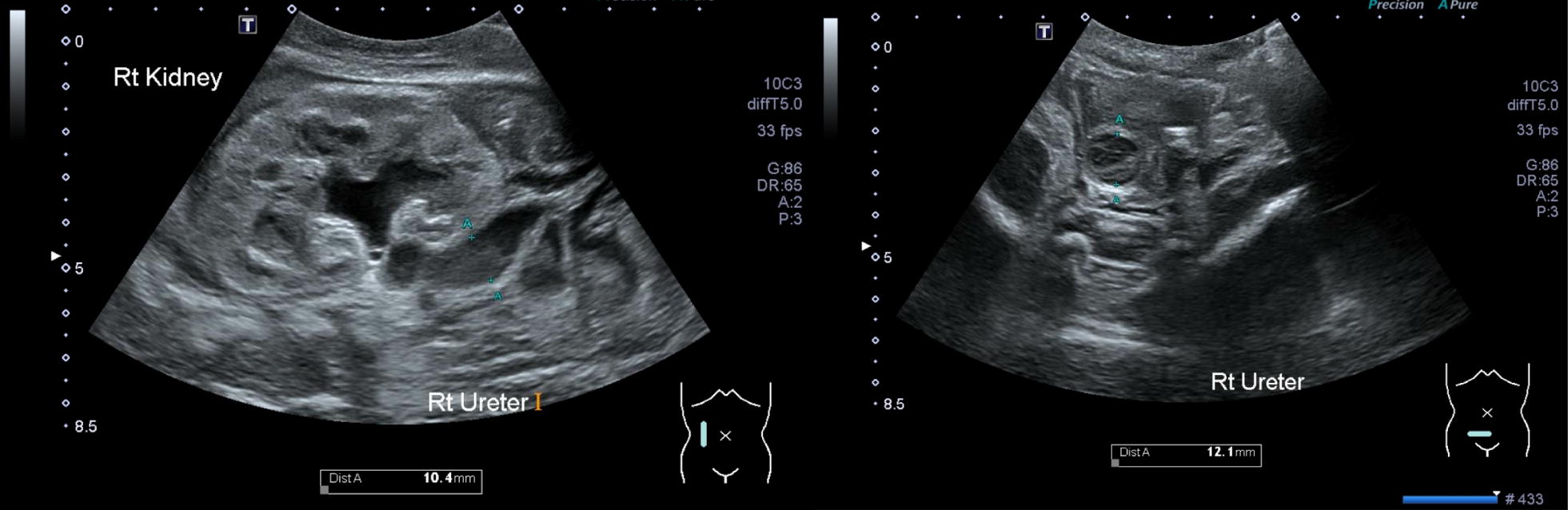
Renal tract ultrasound showing appearance of hydro-nephrosis of right kidney and dilated right ureter (diameter 12.1 mm) on initial presentation. *Rt Kidney* right kidney, *Rt Ureter* right ureter, *Dist A* distance A (12.1 mm)

A 9 day old term neonate presented in septic shock due to methicillin-susceptible *Staphylococcus aureus* urosepsis in the context of severe right-sided hydronephrosis with unilateral pelvic-ureteric and vesico-ureteric junction obstruction (Fig. 1). After a 3 days period on extracorporeal membrane oxygenation, nephrostomy insertion and clinical stabilization, he was completing a 4 week course of IV cephazolin when deteriorated with new fevers and haemodynamic instability. Blood and urine cultures were drawn and empiric vancomycin and gentamicin commenced alongside cephazolin.Rapid identification of positive blood culture broth by our in-house method revealed *Stenotrophomonas maltophilia*, subsequently confirmed on the blood and urine cultures; cerebrospinal fluid cultures were negative. Early identification facilitated timely switch to an appropriate antibiotic regime of single agent intravenous (IV) trimethoprim/sulfamethoxazole (cotrimoxazole) and removal of nephrostomy tube. The child was discharged following a 10 days course of IV cotrimoxazole with full recovery.

### Discussion

This study demonstrates for the first time the utility of rapid identification of positive blood cultures in a pediatric inpatient setting and the impact of this diagnostic approach on clinical decision making.

In this study, we modified a previous protocol [13] to optimize bacterial purity and ease of analysis, including pre-separation of the bacterial pellet from the erythrocytes. The technique was inexpensive, and identification results could be obtained within 15 min of the blood culture flagging positive. Results of the in-house method were highly consistent with single colony identification for Gram-negative organisms to species and genus level (up to 99%), outperforming comparable methods in the literature which reported < 90% concordance [3, 12–16].

Whilst identification had high accuracy for Gram negative bacteria as well as *S. aureus* and *Enterococcus* spp., the method performed less well for other Gram positive organisms and yeasts, consistent with other direct MALDI-TOF MS methodologies [3, 14–16]. The thicker cell walls and spore-forming nature of bacilli [8, 12–16] and the more complex processing required to disrupt yeast cell walls [19] are likely contributors. Some studies, using more complicated and costly extraction procedures, have reported slightly higher success rates for Gram positive organisms [17, 18]. Three out of 14 viridans group Streptococci were misidentified as *S. pneumoniae* due to the inherent difficulty of MALDI-TOF MS in identifying closely related species. This problem has also been documented with the commercial Bruker method [8]. Nevertheless, positive predictive value for *S. pneumoniae* stayed at a higher level (100%) in our assay. Our study was underpowered to adequately assess accuracy for Gram positive anaerobes (e.g. *Clostridium* spp.). Thus, the direct method should be interpreted carefully for potential coagulase-negative staphylococci and Gram-positive bacilli until there is formal identification. Our assay out-performed most published studies [3, 12]; detecting at least one species from 85% of poly-microbial blood cultures with a lowered confidence score. Low organism counts or competition from impurities may explain the lower confidence scores in poly-microbial samples.

There have been very few studies focusing on the impact of rapid identification from MALDI-TOF MS for both the patient and the laboratory [4, 5, 20–24]. Results from our study demonstrate that direct identification of organisms in positive blood culture bottles using MALDI-TOF MS could have important clinical impacts. The potential impact was most significant for the more virulent organisms (*S. aureus, Streptococcus* spp.) and organisms with potential resistance to cephalosporins (*Enterobacter cloacae),* and carbapenems (*Stenotrophomonas maltophilia*) due to required deviation from empiric therapeutic regimens. In patients with central venous access device (CVAD) infections, early identification had clear benefits for prompt CVAD removal. Our results suggested that narrow spectrum therapy could be advised from day one when a common pathogen with predictable susceptibility profile is identified such as *Staphylococcus aureus* or *Enterococcus faecalis*. Early confirmation of contaminated blood cultures was advantageous, leading to potential de-escalation of antibiotics along with complimentary diagnostic testing and shortening of hospital stay, in line with previous studies [2, 5, 22]. Although, clinical impact on infection control was not noteworthy, there was a case of *N. meningitidis* bacteremia which helped to early escalation of infection prevention strategies during the first phase of the study.

We have demonstrated that this technique is highly accurate for Gram negative organisms and *Staphylococcus aureus*, and has potential impacts on length of stay and antibiotic rationalization and infection control decision making. The reliability, rapidity, and simplicity of our technique allow it to be easily adopted by modern microbiology laboratories equipped with MALDI-TOF MS technology.

## Limitation

Our in-house method was incapable of identifying yeast and a randomized controlled study in a larger population would enable assessment of clinical outcomes to a larger degree.

## Data Availability

The datasets used during the current study are available from the corresponding author on reasonable request.

## Abbreviations

MALDI-TOF-MS: matrix assisted laser desorption ionization-time of flight mass spectrometry
PCR: polymerase chain reaction
16s rRNA: 16S ribosomal ribonucleic acid
CVL: central venous line
PPV: positive predictive value
GNR: Gram negative rods
GPR: Gram positive rods
GPCSTA: Gram positive cocci resembling Staphylococci
GPCSTR: Gram positive cocci resembling Streptococci
CLABSI: central venous line-associated bloodstream infection
